# 843. Prognostic Association of Liposomal Amphotericin B Doses Above 5 mg/kg in Mucormycosis: A Nationwide Epidemiologic and Treatment Analysis in Japan

**DOI:** 10.1093/ofid/ofad500.888

**Published:** 2023-11-27

**Authors:** Masato Tashiro, Hotaka Namie, Yuya Ito, Takahiro Takazono, Hiroshi Kakeya, Yoshitsugu Miyazaki, Hiroshi Mukae, Hiroshige Mikamo, Fukuda Tomoo, Kazutoshi Shibuya, Koichi Izumikawa

**Affiliations:** Nagasaki University Graduate School of Biomedical Sciences, Lomita, California; Nagasaki University Graduate School of Biomedical Sciences, Lomita, California; Nagasaki University Hospital, Nagasaki, Nagasaki, Japan; Nagasaki University Graduate School of Biomedical Sciences, Lomita, California; Osaka Metropolitan University, Osaka, Osaka, Japan; National Institute of Infectious Diseases, Shinjuku-ku, Tokyo, Japan; Nagasaki University, Nagasaki, Nagasaki, Japan; Aichi Medical University, Aichi, Aichi, Japan; Saitama Medical Center, Saitama Medical University, Saitama, Saitama, Japan; Toho University Omori Medical Center, Tokyo, Tokyo, Japan; Nagasaki University, Nagasaki, Nagasaki, Japan

## Abstract

**Background:**

Mucormycosis is a fatal fungal infection, and there is limited information on its epidemiology and treatment practices, including the optimal dosage of liposomal amphotericin B.

**Methods:**

A retrospective nationwide analysis of 82 proven and probable cases of mucormycosis was performed. Cases were collected from 51 hospitals in Japan by hematologists and infectious disease specialists. Variables included annual incidence, site of infection, Mucorales genera, and patient and treatment characteristics. Comparison was made between patients who survived and those who died. Comparative analysis was performed between patients who received a liposomal amphotericin B dose of 5 mg/kg and those who received a dose of >5 mg/kg. Categorical variables were tested for differences using Fisher's exact test or χ-squared test. The association between liposomal amphotericin B dose and prognosis was evaluated using the Kaplan-Meier method, and differences were tested using the log-rank test. A Cox proportional hazards model was used to control for confounding.

**Figure 1.**
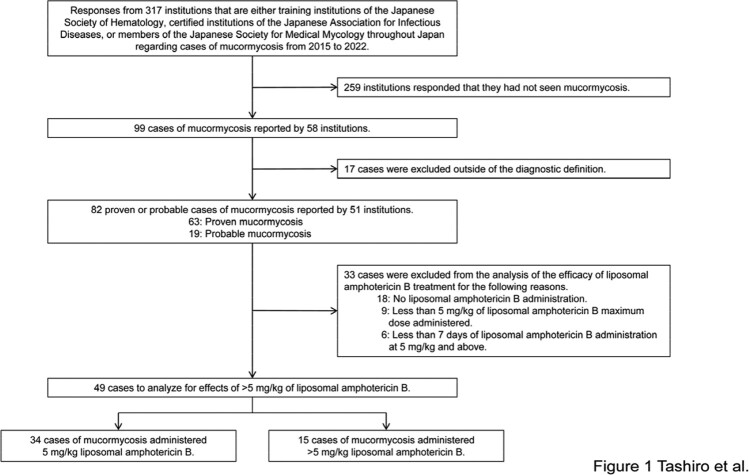
Patient selection

**Results:**

The lung was the most commonly involved organ (70.7%), and 35.4% of cases were disseminated. Rhizopus spp., Cunninghamella spp., and Mucor spp. were the most common organisms. No significant differences in infected organs or distribution of Mucorales were observed between the survival and death groups. Longer duration of neutropenia was more common among deaths (p = 0.007). Mortality within 120 days was 54.2%. More cases in the survival group started liposomal amphotericin B before confirmed diagnosis (p = 0.028). Survival did not differ between patients receiving liposomal amphotericin B at 5 mg/kg and those receiving >5 mg/kg (p=0.873). Using Cox proportional hazards models adjusting for confounders, such as age ( >65 years), prolonged neutropenia, and resection of infected lesions, the hazard ratio for the effect of >5 mg/kg liposomal amphotericin B on 120-day prognosis was 0.93 (95% confidence interval, 0.4-2.3, p = 0.870) compared with 5 mg/kg.

**Figure:**
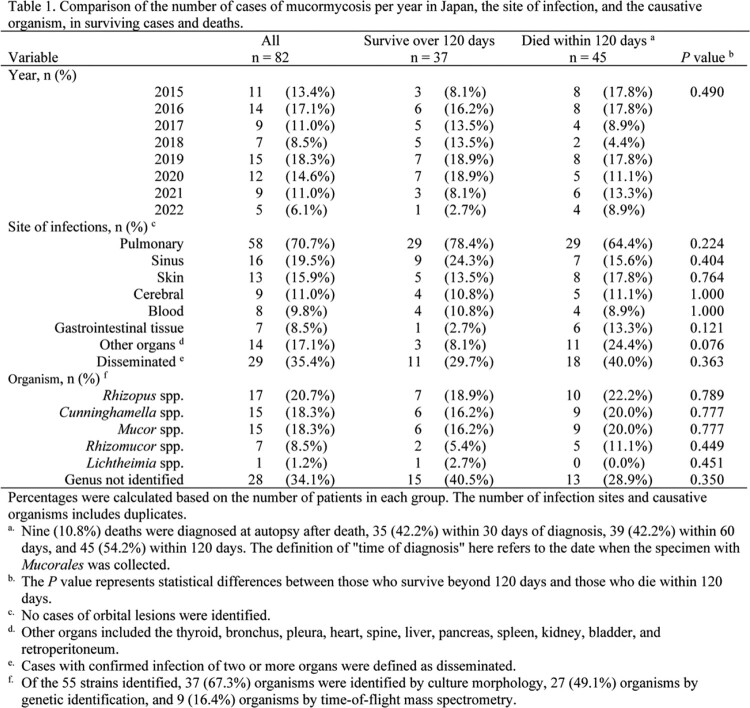

**Figure:**
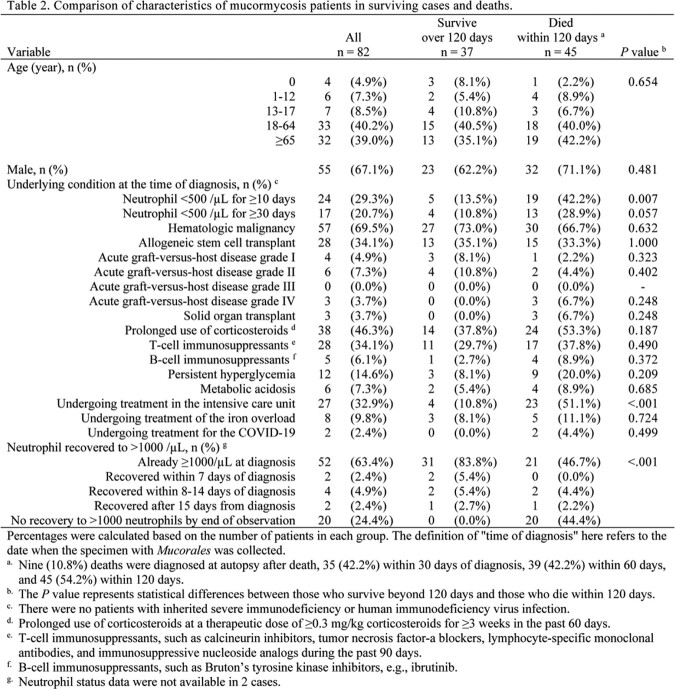

**Figure:**
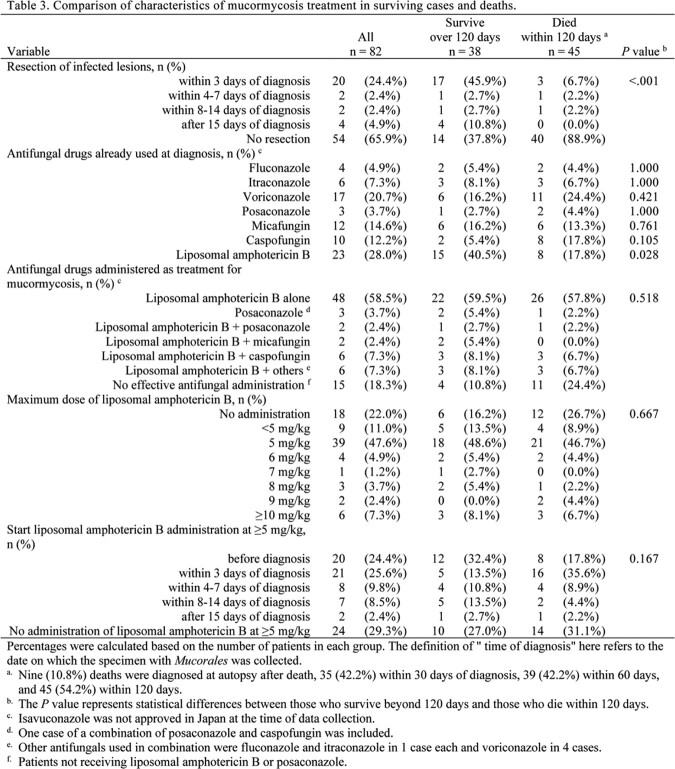

**Conclusion:**

This study provides important insights into the precise epidemiology and treatment practices of mucormycosis among hematologists and infectious disease specialists. There was no difference in prognosis between the 5 mg/kg and >5 mg/kg liposomal amphotericin B groups.

**Figure:**
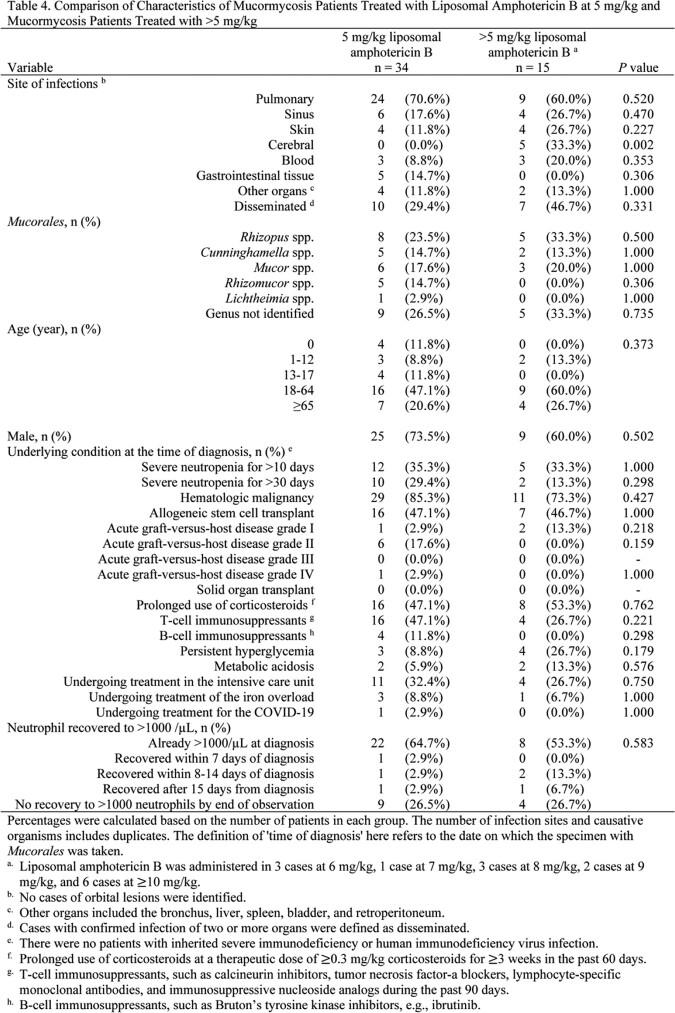

**Figure:**
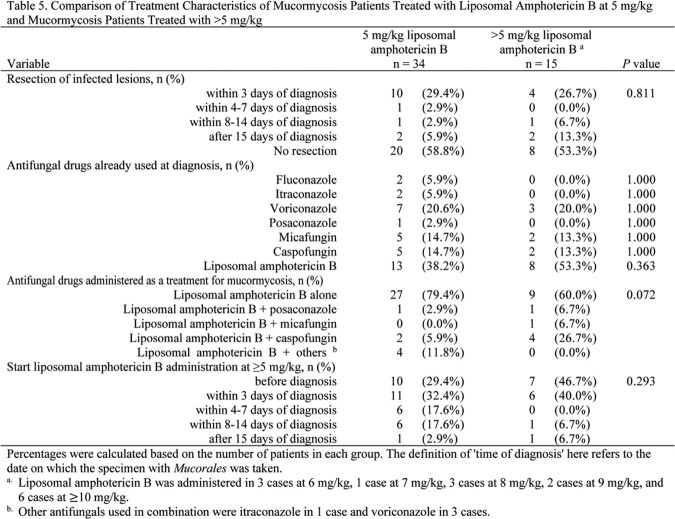

**Figure 2.**
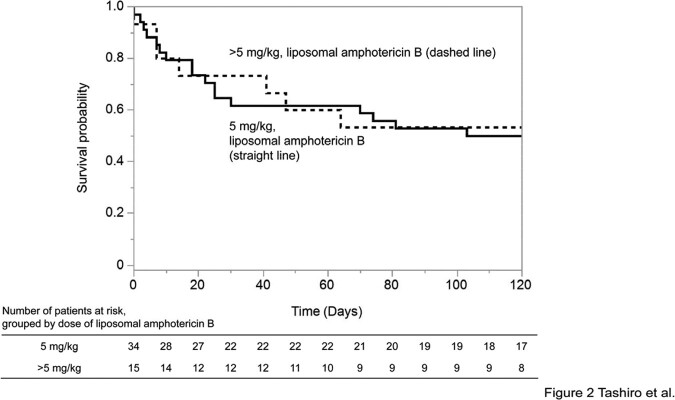
Kaplan-Meier estimates of survival in the two groups of mucormycosis treated with different doses of liposomal amphotericin B.

**Disclosures:**

**Masato Tashiro, MD, PhD**, Asahi Kasei Pharma Corporation: Advisor/Consultant|Asahi Kasei Pharma Corporation: Honoraria|Sumitomo Pharma Co., Ltd.: Honoraria **Hiroshi Kakeya, MD, PhD**, Asahi Kasei Pharma Corporation: Honoraria|Merck Sharp & Dohme: Honoraria|Sumitomo Pharma Co., Ltd.: Honoraria **Hiroshige Mikamo, M.D, Ph.D**, Asahi Kasei Pharma Corporation: Grant/Research Support|Merck Sharp & Dohme: Honoraria|Pfizer Inc.: Grant/Research Support|Pfizer R&D Japan: Honoraria|Sumitomo Pharma Co., Ltd.: Grant/Research Support|Sumitomo Pharma Co., Ltd.: Honoraria **Kazutoshi Shibuya, M.D., Ph.D.**, Sumitomo Pharma Co., Ltd.: Grant/Research Support **Koichi Izumikawa, M.D., Ph.D.**, Asahi Kasei Pharma Corporation: Grant/Research Support|Asahi Kasei Pharma Corporation: Honoraria|Astellas Pharma Inc.: Honoraria|DAIICHI SANKYO COMPANY, LIMITED: Grant/Research Support|DAIICHI SANKYO COMPANY, LIMITED: Honoraria|KYORIN Pharmaceutical Co., Ltd.: Honoraria|Merck & Co., Inc.: Honoraria|Pfizer Japan Inc.: Honoraria|Shionogi & Co., Ltd.: Grant/Research Support|Shionogi & Co., Ltd.: Honoraria|Sumitomo Pharma Co., Ltd.: Grant/Research Support|Sumitomo Pharma Co., Ltd.: Honoraria|TAIHO PHARMACEUTICAL CO., LTD.: Grant/Research Support

